# Predicting the Naturalistic Course of Major Depressive Disorder Using Clinical and Multimodal Neuroimaging Information: A Multivariate Pattern Recognition Study

**DOI:** 10.1016/j.biopsych.2014.11.018

**Published:** 2015-08-15

**Authors:** Lianne Schmaal, Andre F. Marquand, Didi Rhebergen, Marie-José van Tol, Henricus G. Ruhé, Nic J.A. van der Wee, Dick J. Veltman, Brenda W.J.H. Penninx

**Affiliations:** a Department of Psychiatry and Neuroscience Campus Amsterdam, VU University Medical Center Amsterdam, Amsterdam, The Netherlands; b Donders Institute for Brain, Cognition and Behaviour, Radboud University, Nijmegen, The Netherlands; c Department of Neuroimaging, Institute of Psychiatry, King’s College London, United Kingdom; d Department of Psychiatry and the EMGO^+^ Institute for Health and Care Research, VU University Medical Center Amsterdam, Amsterdam, The Netherlands; e Neuroimaging Center, University Medical Center Groningen, University of Groningen, Groningen, The Netherlands; f Department of Psychiatry, Mood and Anxiety Disorders, University Medical Center Groningen, University of Groningen, Groningen, The Netherlands; g Department of Psychiatry (NJvdW), Leiden University Medical Center, Leiden University, Leiden, The Netherlands

**Keywords:** Clinical information, Course trajectory, Magnetic resonance imaging, Major depressive disorder, Prediction, Probabilistic pattern recognition analysis

## Abstract

**Background:**

A chronic course of major depressive disorder (MDD) is associated with profound alterations in brain volumes and emotional and cognitive processing. However, no neurobiological markers have been identified that prospectively predict MDD course trajectories. This study evaluated the prognostic value of different neuroimaging modalities, clinical characteristics, and their combination to classify MDD course trajectories.

**Methods:**

One hundred eighteen MDD patients underwent structural and functional magnetic resonance imaging (MRI) (emotional facial expressions and executive functioning) and were clinically followed-up at 2 years. Three MDD trajectories (chronic *n* = 23, gradual improving *n* = 36, and fast remission *n* = 59) were identified based on Life Chart Interview measuring the presence of symptoms each month. Gaussian process classifiers were employed to evaluate prognostic value of neuroimaging data and clinical characteristics (including baseline severity, duration, and comorbidity).

**Results:**

Chronic patients could be discriminated from patients with more favorable trajectories from neural responses to various emotional faces (up to 73% accuracy) but not from structural MRI and functional MRI related to executive functioning. Chronic patients could also be discriminated from remitted patients based on clinical characteristics (accuracy 69%) but not when age differences between the groups were taken into account. Combining different task contrasts or data sources increased prediction accuracies in some but not all cases.

**Conclusions:**

Our findings provide evidence that the prediction of naturalistic course of depression over 2 years is improved by considering neuroimaging data especially derived from neural responses to emotional facial expressions. Neural responses to emotional salient faces more accurately predicted outcome than clinical data.

Major depressive disorder (MDD) is worldwide among the leading causes of disability (1) due to high prevalence, negative impact on quality of life, and its frequently recurrent or chronic character. Of all MDD patients, 20% to 25% are at risk for chronic MDD (2). Identifying predictors of chronicity at an early stage is of critical importance, because it can help to select treatment strategies specifically aimed at reducing factors associated with worse long-term clinical outcome.

In MDD, several clinical characteristics have been linked to a chronic course, including greater symptom severity, longer duration of an episode, number of episodes, comorbidity, earlier onset, childhood adversity, higher neuroticism, lower extraversion, and lower conscientiousness (2–7). However, these factors do not directly relate to underlying pathophysiological mechanisms and cannot fully capture interindividual differences in the course of MDD. It is essential to identify additional pathophysiological markers to guide treatment selection and eventually develop alternative treatment strategies. Neuroimaging might provide such biomarkers. On a structural level, reduced hippocampus and anterior cingulate cortex (ACC) volume may represent a vulnerability factor for poor outcome (8,9). On a functional level, aberrant activation related to emotional and cognitive processing (including executive functions) has been implicated (10). For example, alterations in activation in medial prefrontal regions including the ACC during processing of emotional stimuli predict relapse after 18 months in remitted MDD patients (11) and treatment response (12). In addition, abnormal dorsolateral prefrontal cortex (PFC) recruitment during visuospatial planning is related to a nonfavorable naturalistic course of MDD (Woudstra S, *et al.*, unpublished data, 2014). These neuroimaging findings, however, are based on group comparisons with unknown translational value. To make these results clinically useful, it is necessary to provide valid predictions at the level of the individual patient.

Multivariate pattern recognition (MPR) methods have been applied to neuroimaging data to classify individuals as MDD patients or control subjects (13–19). MPR is a technique that allows classification of individuals into distinct classes based on high-dimensional data and is more sensitive for detecting spatially distributed effects, compared with univariate approaches, which aim to detect functionally localized differences.

These diagnostic MPR studies are an important first step, but the real potential of MPR is for predicting future outcome, such as treatment response or course trajectory. To date, only a few preliminary MPR studies have examined whether outcome can be predicted, showing accuracies of 65% to 89% (17,20–22). These studies all focused on small clinical samples of MDD patients recruited in specialized mental health care. Therefore, they capture patients with the most severe and recurrent MDD, who are more likely to be referred to specialized mental health care (23) and who represent only a small proportion of the spectrum of MDD patients. Because most MDD patients reside in the community and primary care, the generalizability of these MPR findings to a general population remains unclear. It is of great clinical relevance to predict the course of MDD in a sample derived from a more naturalistic setting where patients have a broad range of illness severity. Moreover, MPR studies to date have mostly focused on a single imaging modality. It is unknown which imaging modality or functional task provides the most accurate predictions of outcome. Finally, little is known about the added value of neuroimaging to predict MDD disease course relative to cheaper and more easily acquired measures such as clinical assessments.

The current aim was to employ MPR to identify predictors for chronicity of MDD. For this purpose, we employed Gaussian process classifiers (GPCs) to examine the potential of various imaging modalities including structural magnetic resonance imaging (MRI) and brain activity during emotional and cognitive processing. In addition to these imaging modalities known important clinical variables, such as baseline severity, duration, and comorbidity indicators and information on personality traits and childhood trauma, were used to discriminate between different MDD course trajectories in 118 individual patients with a current MDD diagnosis from a naturalistic cohort encompassing the broad heterogeneity of MDD.

## Methods And Materials

### Subjects

After approval of the NEtherlands Study of Depression and Anxiety (NESDA)-MRI study by the ethical review boards of the three participating centers and written informed consent of participants, a subgroup (total *n* = 301; subjects with MDD diagnosis *n* = 156) of participants from the total NESDA study was included for MRI. Of these, for the current study, we included all 118 patients (82 female patients; aged 18–56) who had 1) baseline current (6-month) DSM-IV diagnosis of MDD, established using the structured Composite International Diagnostic Interview (24) and reporting symptoms in the month before baseline confirmed with either the Composite International Diagnostic Interview or the Life Chart Interview (LCI) (25); and 2) availability of 2-year follow-up of depressive symptoms measured with the LCI.

### Definition of Two-Year Course Trajectory Groups

Based on a latent class growth analysis (LCGA) of follow-up data derived from the LCI [which was the source containing most detailed information on 2-year MDD course, previously conducted in a larger, overlapping sample (7)], MDD patients were divided in different course trajectories. Briefly, LCGA analysis, based on the burden of depressive LCI symptoms indicated for each of the 24 months between baseline and follow-up (with the first score representing the burden of symptoms in the month after baseline) was conducted in 804 MDD patients. The LCGA analysis identified five different classes of course trajectories: 1) a rapid remission trajectory; 2) a trajectory showing a gradual improvement of symptoms; 3) a second trajectory showing a gradual improvement of symptoms but with higher initial depressive symptom scores; 4) a chronic trajectory with moderate initial severity; and 5) a chronic trajectory with severe initial severity. Because the two improving trajectories, as well as the two chronic trajectories, were very similar and for the purpose of increasing power, we combined these pairs, yielding three course trajectories: 1) MDD-remitted (REM), showing a rapid remission of symptoms (*n* = 59); 2) MDD-improved (IMP), showing a gradual improvement in symptoms from baseline to follow-up (*n* = 36); and 3) MDD-chronic (CHR), showing no relief from symptoms from baseline to follow-up (*n* = 23). See Figure S1 in Supplement 1 for a graphic representation of these symptom trajectories. We emphasize that although these class labels were determined on an overlapping sample, the measures employed to predict them were distinct, thereby avoiding circularity.

### Baseline Clinical Predictors

The prognostic value of several baseline clinical characteristics was assessed, including severity of depression using the Inventory of Depressive Symptomatology (IDS) (26), severity of anxiety using the Beck Anxiety Inventory (27), information on duration of depressive and anxiety symptoms before baseline derived from the baseline LCI (assessing the number of months the patient spent with depressive and/or anxiety symptoms 4 years before baseline), age of onset, and years since first episode, plus neuroticism, extraversion, and conscientiousness personality traits from the corresponding scales of the NEO-Five Factor Inventory questionnaire (28). Additionally, childhood trauma (before age 16) was measured by structured interview and indexed from 0 to 8, as used previously (29). These measures to predict MDD course were all independent from the measure that was used to define the course trajectory groups (i.e., burden of depressive symptom scores derived from the LCI, which was assessed at 2-year follow-up).

### Functional MRI Task Paradigms

**Faces Task.** An emotional faces paradigm was used to assess brain activation during emotion processing. Color pictures of angry, fearful, sad, happy, and neutral facial expressions, plus a control condition consisting of scrambled faces, from the Karolinska Directed Emotional Faces System (30) were presented. Contrasts used to train the classifier were angry > scrambled faces, fearful > scrambled faces, happy > scrambled faces, and sad > scrambled faces. See Supplement 1 for details.

**Tower of London Task.** A Tower of London (ToL) task was used to assess brain activity during visuospatial planning. Contrast images for task load were used to train the classifier. See Supplement 1 and van Tol *et al.* (31) for details.

### Image Acquisition

Magnetic resonance imaging data were obtained using 3T Phillips MRI scanners (Phillips Healthcare, Best, The Netherlands) located at the three participating centers, equipped with a SENSE 8-channel (Leiden University Medical Center and University Medical Center Groningen) and a SENSE 6-channel (Academic Medical Center) receiver head coil (Phillips Healthcare). See Supplement 1 for details.

### Data Analysis

**Clinical Characteristics.** Each subject’s scores on the IDS, Beck Anxiety Inventory, NEO-Five Factor Inventory, number of months with depressive symptoms before baseline, number of months with anxiety symptoms before baseline, age of MDD onset, years since first episode, and a childhood trauma index were concatenated and this matrix was used as input to GPCs.

**Image Processing of MRI Data.** T1 images were normalized and segmented into gray matter, white matter, and cerebrospinal fluid using the voxel-based morphometry toolbox (VBM8; http://dbm.neuro.uni-jena.de/vbm.html) and functional images were preprocessed and analyzed with statistical parametric mapping (SPM) (SPM8; http://www.fil.ion.ucl.ac.uk/spm/software/). For each functional MRI (fMRI) task, samples for the classifier were constructed by estimating a general linear model (32). See Supplement 1 for full details.

### Pattern Recognition Analysis

We applied binary GPCs, as implemented in the Pattern Recognition for Neuroimaging Toolbox (33) (http://www.mlnl.cs.ucl.ac.uk/pronto), to investigate the potential of whole-brain structural and functional images and clinical characteristics for predicting the naturalistic course of MDD. GPCs are a supervised MPR approach similar to support vector machines that provide the added benefit of predictive probabilities of class membership. For details, see Marquand *et al.* (34). For each modality, independent binary GPCs were used to discriminate different trajectories. To assess generalizability, each GPC was repeatedly retrained with leave-one-out cross-validation, where all data from a single subject were excluded at each iteration. For each subject, the GPC provided probabilistic predictions for each trajectory, which were converted to categorical predictions by applying a threshold according to the frequency of classes in the training set (i.e., .5 if the classes are balanced). Since some of the classifiers were unbalanced (i.e., with one class being larger than the other), balanced accuracy measures (the mean of sensitivity and specificity) were computed to assess the overall categorical performance of each classifier in a way that accommodated this imbalance. Statistical significance was determined by permutation testing; the whole cross-validation cycle was repeated for each permutation and the labels of the training data were permuted across subjects (34); see Supplement 1 for full details. In addition, a label fusion technique was applied to combine all data modalities (Supplement 1). For each modality and contrast, *p* values were corrected for multiple comparisons using the Benjamini and Hochberg step-up method (35).

For clinical applications, an important advantage of probabilistic classifiers is the ability to identify cases where the classifier does not provide a confident prediction of trajectory. In such cases, a reject option (36) may be specified, where the final decision is deferred to a clinician. We explore the use of such a reject option for all classifiers exceeding chance by smoothly varying the rejection threshold and computing the accuracy, leading to an accuracy-reject curve (37,38).

### Predictive Maps

To characterize the discriminative pattern across brain regions, we employed a simple method that provides coefficients that can be interpreted in terms of the pattern of effects across brain regions (39) and compared this approach with mass-univariate SPM. See Supplement 1 for details.

## Results

### Sample Characteristics

Course trajectories did not differ with regard to gender, years of education, scan location, baseline antidepressant use, or follow-up (Table 1). However, trajectories differed in age (*F*_2,115_ = 4.92, *p* = .01), with CHR subjects being older than both REM (*t*_80_ = 2.89, *p* < .005) and IMP subjects (*t*_57_ = 2.84, *p* = .01). Therefore, to control for the potential confounding effect of age, analyses were repeated with every subject in the CHR group (*n* = 23) matched with an equivalent with respect to age, gender, and education in the REM (*n* = 23) and IMP (*n* = 23) groups (reported in Supplement 1). As an additional validation of our definition of the different course trajectory groups, which were defined on the basis of the LCI burden of symptom scores, IDS scores assessed at baseline interview, baseline scanning, and 2-year follow-up were compared between the three groups. Course trajectory groups did not differ with regard to IDS scores both at baseline interview (Table 1) and at time of baseline scanning (Supplement 1). As would be expected on the basis of the different depression courses, the groups differed on IDS scores at 2-year follow-up (*F*_2,115_ = 13.22, *p* < .001), with depression severity scores being higher in the CHR than REM subjects (*t*_80_ = 12.66, *p* < .001) and IMP subjects (*t*_57_ = 7.53, *p* = .005) (Table 1).

Of all 118 subjects, for the faces task, fMRI data from 20 REM, 5 IMP, and 8 CHR patients were discarded because of having performed a different (noncomparable) version of the task, inferior data quality, or incomplete coverage of the temporal lobe (final sample faces task: REM *n* = 39, IMP *n* = 31, and CHR *n* = 15), and for the ToL task, fMRI data from 5 REM and 4 CHR patients were discarded because of inferior data quality, incomplete coverage of the temporal lobe, or poor performance (overall proportion correct responses <75%) (final sample ToL task: REM *n* = 54, IMP *n* = 36, and CHR *n* = 19).

### Gaussian Process Classification Using Clinical Characteristics

Using baseline clinical information, the GPC discriminated between the CHR and REM subjects (Table 2) but not between CHR and IMP subjects or between IMP and REM subjects.

### Gaussian Process Classification Using Faces Task Contrast Images

**Chronic Versus Remitted Patients.** The GPCs for angry > scrambled faces and happy > scrambled faces accurately discriminated between CHR and REM subjects (Table 2). The GPC for fearful > scrambled faces also discriminated classes but did not survive multiple comparison correction. When combining the five different emotional conditions, the GPC discriminated between the CHR and the REM subjects with the highest accuracy obtained by any contrast (73%).

**Figure 1:**
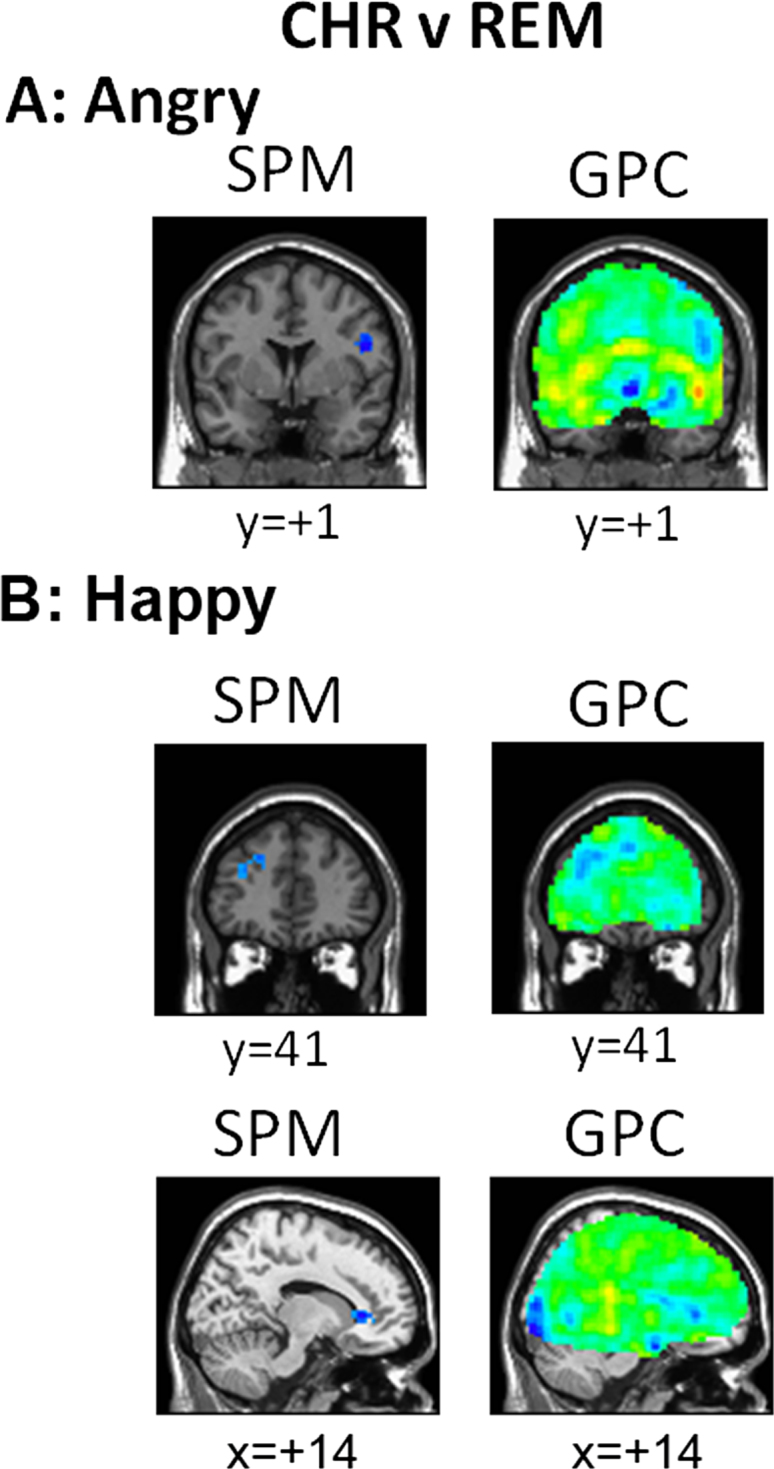
Gaussian process classifier (GPC) predictive maps for discriminating major depressive disorder (MDD)-chronic (CHR) and MDD-remitted (REM) subjects. Representative slices from GPC predictive maps discriminating MDD-CHR from MDD-REM subjects plus statistical parametric maps (SPMs) thresholded at *p* < .001, presented separately for the contrasts **(A)** angry versus scrambled faces and **(B)** happy versus scrambled faces. The red colors indicate higher prognostic value for the first class (i.e., MDD-CHR) and blue colors indicate voxels with a higher prognostic value for the second class (MDD-REM).

Representative slices from the patterns discriminating CHR from REM subjects are shown in Figure 1A–D and whole-brain images in Supplement 1. These patterns are by nature dense in that they have nonzero coefficients in every brain region. However, the highest coefficients showed a strong correspondence to the regions showing focal group differences in the SPM. In all regions, these indicate reduced activity in CHR subjects. Highest coefficients favored REM relative to CHR subjects and were found in dorsolateral prefrontal cortex for the angry > scrambled contrast and in medial and dorsolateral PFC for happy > scrambled (Figures 1A–C).

**Figure 2:**
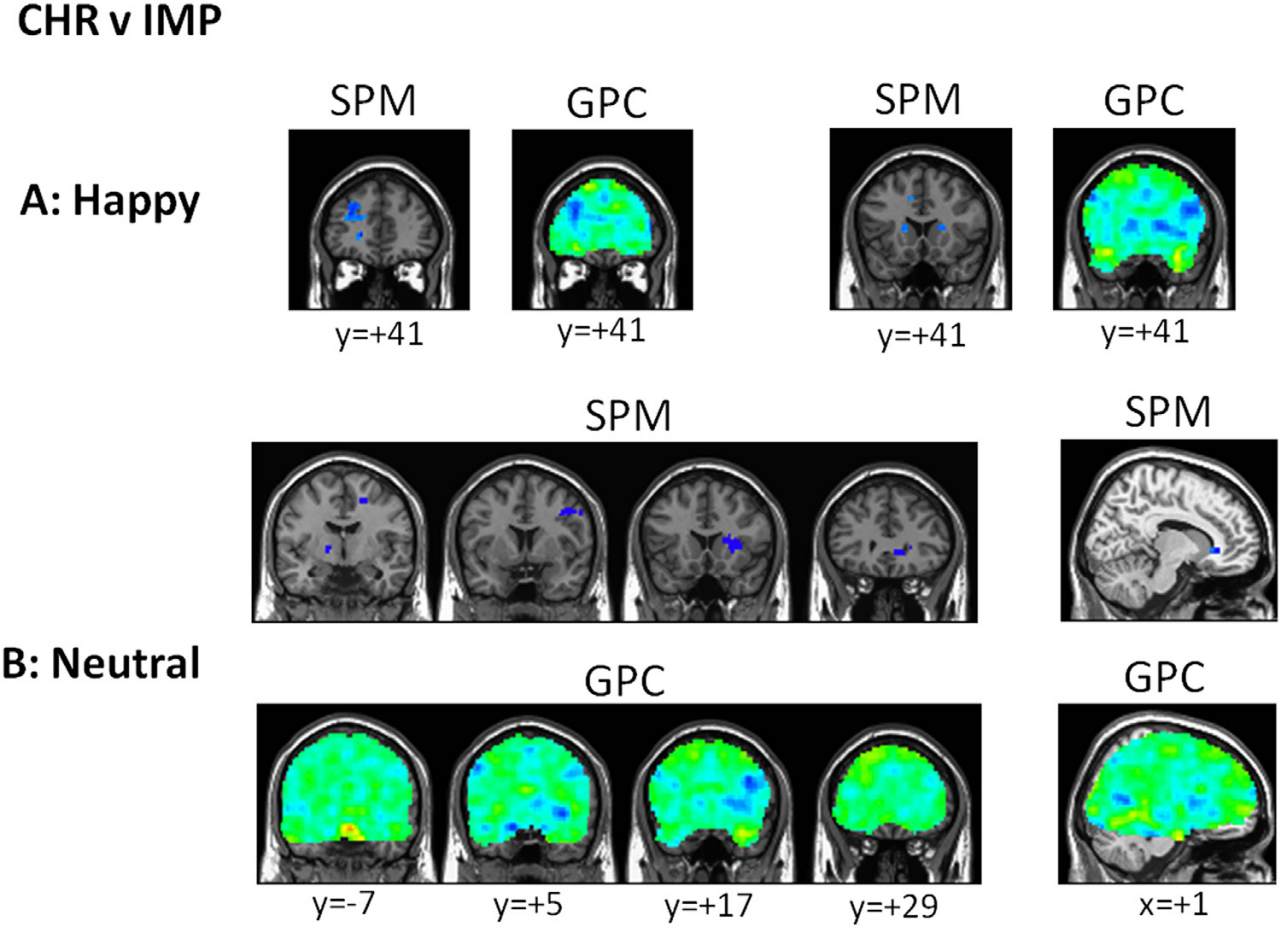
Gaussian process classifier (GPC) predictive maps for discriminating major depressive disorder (MDD)-chronic (CHR) and MDD-improvement (IMP) subjects and MDD-IMP and MDD-remitted (REM) subjects. Representative slices from GPC predictive maps discriminating MDD-CHR from MDD-IMP subjects and statistical parametric maps (SPMs) (thresholded at *p* < .001) presented separately for the contrasts **(A)** happy versus scrambled faces and **(B)** neutral versus scrambled faces. The red colors indicate higher prognostic value for the first class (i.e., MDD-CHR) and blue colors indicate voxels with a higher prognostic value for the second class (MDD-IMP).

**Chronic Versus Gradual Improvement in Symptoms Patients.** Chronic subjects could be distinguished from the IMP subjects on the basis of patterns of neural activity for happy > scrambled faces and neutral > scrambled faces (Table 2). The correspondence of the patterns discriminating CHR from IMP subjects with the SPM was again high and activity was again reduced in CHR subjects. High coefficients favoring IMP relative to CHR were found in the dorsolateral PFC and bilateral caudate for happy faces and in medial and dorsolateral PFC plus the basal ganglia for neutral faces (Figure 2).

**Gradual Improvement in Symptoms Versus Remitted Patients.** The IMP and REM groups could not be discriminated on basis of patterns of neural activity for any of the emotional facial expressions (Table 2).

**Gaussian Process Classification Using Other Neuroimaging Modalities.** None of the course trajectories could be discriminated using either patterns of neural activity in response to increasing task load of the ToL or gray matter images (Table 2).

### Combining Classifiers from Different Modalities

Using a combination of all information, including clinical, structural MRI, and fMRI data, the GPC discriminated between CHR and REM subjects (Table 2). The combined classifier was not able to distinguish between CHR and IMP subjects and between REM and IMP subjects. As both patterns of neural activity elicited by emotional faces as well as clinical data were individually able to predict depression course for CHR versus REM subjects, we examined whether combining only these modalities would improve prediction accuracy (Table 2). For all contrasts, this resulted in lower accuracy relative to either modality separately.

### Results Using Groups Matched on Age

The accuracies obtained when subjects were matched on age are provided in Supplement 1. These were highly similar to the nonmatched sample results, albeit with generally higher accuracy.

### Assessment of Predictive Confidence

**Figure 3:**
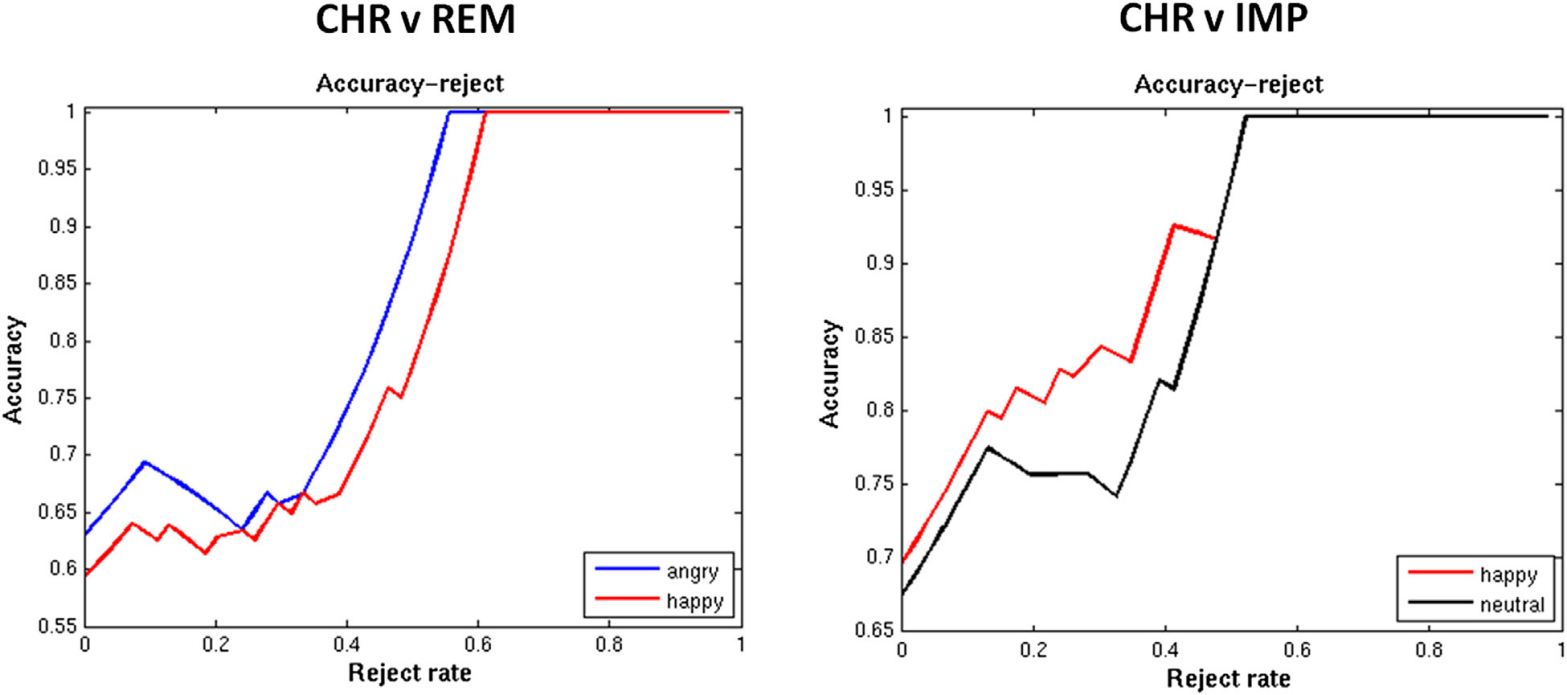
Accuracy-reject curves for the classifiers exceeding chance. Accuracy-reject curves for classifiers exceeding chance that discriminated **(A)** major depressive disorder (MDD)-chronic (CHR) from MDD-remitted (REM) subjects and **(B)** MDD-CHR from MDD-improvement (IMP) subjects. The accuracy-reject curve illustrates the accuracy of the classifier when only predictions greater than a certain confidence threshold are considered (e.g., above .6). Cases that do not meet this threshold can then be deferred to a clinician or other decision support system. This is known in the pattern recognition literature as adopting a reject option. The curve is constructed by smoothly varying the decision threshold computing the accuracy at each stage.

We show accuracy-reject curves for all classifiers that exceeded chance. These clearly show that if one is prepared to accept a reject option, then accuracy can be improved significantly. For example, at a rejection threshold of 60% of subjects, perfect classification can be achieved with all classifiers considered (Figure 3).

## Discussion

We employed probabilistic MPR to predict the future course of MDD—at the level of individual subjects—in a naturalistic cohort encompassing the broad heterogeneity of MDD. A chronic trajectory could be accurately discriminated with maximum accuracies of 1) 73% for discriminating subjects with a chronic course from those that showed a rapid remission (CHR versus REM); and 2) 69% for discriminating subjects with a chronic course from those showing a gradual improvement in symptoms (CHR versus IMP). The neurobiological markers that discriminated each contrast were distinct. For CHR versus REM, subjects could be discriminated based on neural responses to angry and happy facial expressions but not on structural MRI or neural correlates of executive functioning. In contrast, for CHR versus IMP, subjects could be discriminated based on neural responses to happy and neutral expressions. CHR subjects could be differentiated from REM subjects based on a combination of clinical variables; however, this was probably driven by differences in age, as the accuracy became nonsignificant when groups were matched on age. Accuracies based on neural responses to emotional facial expressions showed a similar pattern in the smaller matched sample as in the full sample but were higher overall, indicating 1) a robust prediction of naturalistic course of MDD using neuroimaging data related to emotional processing; and 2) that the confounding effect of age was to impair classification, not to assist it.

In the present study, it is especially noteworthy that 1) the clinical measures were poorer predictors of outcome than the neurobiological measurements; and 2) that the visuospatial planning task and structural neuroimaging measures were not discriminative, which corresponds with previous reports of fMRI providing more accurate predictions than structural MRI (40). In contrast to these findings, previous studies, including our own work in an overlapping NESDA sample, have indicated an association between worse long-term outcome and these baseline clinical characteristics (2–7). In addition, our previous work using mass-univariate regression showed a relation between focal abnormal dorsolateral prefrontal cortex recruitment during visuospatial planning and a nonfavorable naturalistic course of MDD (Woudstra S, *et al*., unpublished data, 2014). Moreover, reduced hippocampus and ACC volume have been associated with poor outcome (41). However, these findings were all based on group-wise associations, and although these baseline clinical and neuroimaging parameters can be associated with outcome based on group-level (mass-univariate) approaches, they might not possess sufficient prognostic ability for long-term outcome in individual patients, as observed in the current study using MPR methods.

Neural measures of affective processing showed higher prognostic ability than gray matter volumes and patterns of neural activity during executive functioning, which is in line with earlier MPR work indicating that implicit processing of sad facial affect provided more accurate diagnostic predictions than a working memory task in the same subjects (17,42). This observation together with evidence of aberrant emotion-regulation processing in MDD (43) suggest that 1) affective processing deficits are at the basis of MDD; and 2) CHR subjects comprise a more distinguishable subgroup of MDD patients than other trajectories. The problem of predicting MDD disease course is highly challenging but has direct clinical relevance because identifying patients likely to have a chronic course early in the disease process can help clinicians to target interventions more effectively (41). Several studies have demonstrated the potential of MPR for predicting the presence of an MDD diagnosis, but only a few studies have demonstrated its utility for MDD prognosis (44). An important contribution of the present work is to demonstrate the utility of MPR for predicting outcome in a naturalistic setting where the depressive phenotype is simultaneously less severe, more heterogeneous, and more reflective of the variability in the MDD phenotype than the cohorts studied to date.

This study aimed to discriminate subjects based on distributed patterns of neural activity, which is a complementary objective to identifying focal brain characteristics associated with disease trajectory. The pattern in our study that discriminated between chronic subjects and those with more favorable trajectories on the basis of brain activation in response to sad, fearful, angry, and neutral facial expressions showed high coefficients in regions that also showed focal differences and included regions that did not survive mass univariate thresholding, where the effects may have been more subtle but nevertheless still predictive of outcome in the context of the multivariate pattern. The most important regions for predicting favorable, relative to chronic disease, courses included dorsolateral and medial prefrontal regions, striatum, and parietal regions, all of which have been strongly implicated in the neurobiology of processing emotional stimuli, depression, and treatment response (42,45,46).

Although we expected improved accuracy by using different modalities, the accuracies obtained by the multi-modal classifiers combining different neuroimaging modalities and baseline clinical information did not produce improvements in accuracy for predicting different course trajectories. In contrast, the classifier combining all emotional faces task conditions yielded a 9% improvement over the most discriminative task condition for the CHR versus REM contrast and produced the highest accuracy of any modality. These results indicate that fusing different data sources is probably most beneficial if a substantial proportion of them are individually and independently predictive. In other words, combining sources that are not predictive individually with sources that are predictive only increases noise and reduces the ability of the classifier to discriminate groups. An important line of future work is to determine whether combining neuroimaging and clinical information with additional sources like neuropsychological or biochemical tests would improve accuracy. Another line of future work is to generalize beyond binary classification. Here, we aimed to demonstrate that disease trajectories could be discriminated from one another, for which a binary classifier is suitable. However, this procedure might limit the inferences we can draw for new cases in clinical practice. Using the current approach, we can infer whether a new case is, for example, more likely to remit than show a chronic course or more likely to be chronically depressed than improve over time but not derive a single class prediction. Therefore, a multi-class classifier more closely matches the clinical decision process and will be investigated in follow-up studies.

Previous MPR studies in depression have reported accuracies in the range of 67% to 86% for diagnosis and 68% to 89% for predicting treatment response (cognitive behavioral therapy or medication) (44). The accuracies we report are within this range, even though the problem of predicting naturalistic outcome is considerably more difficult than predicting diagnosis or the outcome of a controlled intervention because 1) this cohort is highly heterogeneous, encompassing a range of depressive phenotypes from very mild to severe; and 2) the treatments the patients received were not standardized. We argue that it is precisely for this reason that this work furnishes an important transition toward real-world clinical populations, including MDD patients recruited from community and primary care settings where the majority of MDD patients reside and where patients have a broad range of illness severity.

### Limitations

A limitation is that we combined two groups showing gradual improvement and the two chronic groups from the five trajectories that were identified by the previous LCGA analysis (7). Although the trajectories were not different between either the original two improvement or chronic groups, they were dissociable in their baseline burden score. However, by assuring that the groups in the current study did not differ in their initial burden score, we can be more confident that our findings truly reflect the prognostic value and not merely different baseline severities. Another limitation of our study relates to the cross-validation approach we employed to assess generalizability. While cross-validation is known to be an approximately unbiased estimator (47) of population generalizability, it may not completely account for the different characteristics of data from different samples [e.g., scanner effects, see (48)]. An important next step is to validate the classification models in completely independent data.

### Conclusion

The current study clearly showed that prediction of naturalistic course of MDD is possible using neuroimaging data. Moreover, this approach provided more accurate indicators of outcomes than predictions based on clinical data only. Our results indicate patterns of abnormalities that can distinguish different course trajectories and pave the way for the development of decision support tools that can be used in clinical practice.

## Figures and Tables

**Table 1:** Demographic and Clinical Characteristics of Subjects Included in the MVPA Analyses

Characteristic	MDD-REM (*n* = 59)	MDD-IMP (*n* = 36)	MDD-CHR (*n* = 23)	Statistic	*p* Value
Age, Years	35.58 (10.53)	35.59 (9.56)	43.00 (10.24)	*F* = 4.92	.01^a^
Gender, *n* (%)					
Female	44 (75)	25 (68)	13 (56)	χ^2^ = 2.56	.28
Male	15 (25)	12 (32)	10 (44)		
Education, Years	12.31 (3.50)	11.97 (3.03)	12.48 (2.54)	*F* = .21	.81
Scan Location, *n* (%)					
AMC Amsterdam	18 (30)	9 (25)	9 (39)	χ^2^ = 2.94	.57
LUMC Leiden	18 (30)	16 (43)	8 (35)		
UMCG Groningen	23 (40)	12 (32)	6 (26)		
IDS Total T1	31.58 (10.51)	32.61 (9.88)	35.78 (8.28)	*F* = 1.49	.23
IDS Total T2	17.03 (10.35)	21.76 (9.95)	29.70 (10.13)	*F* = 13.22	<.001^b^
IDS Change (T2 − T1)	−14.55 (13.11)	−10.44 (11.23)	−6.08 (9.82)	*F* = 4.38	.02^c^
Antidepressant Use T1, *n* (%)					
No	38 (64)	26 (70)	14 (61)	χ^2^ = .62	.73
Yes	21 (36)	11 (30)	9 (39)		
Antidepressant Use T2, *n* (%)					
No	37 (63)	26 (70)	15 (65)	χ^2^ = .58	.75
Yes	22 (37)	11 (30)	8 (35)		
Duration of Use of Antidepressants between Baseline and Follow-up (Including Currently Used at Follow-up), Months	20.37 (38.11)	16.31 (32.30)	13.00 (23.73)	*F* = .43	.65

Data are given as mean (SD). AMC, Academic Medical Center; IDS, Inventory of Depressive Symptoms; LUMC, Leiden University Medical Center; MDD-CHR, major depressive disorder chronic group; MDD-IMP, major depressive disorder gradual improvement in symptoms group; MDD-REM, major depressive disorder remitted group; MPR, multivariate pattern recognition; T1, baseline; T2, 2-year follow-up; UMCG, University Medical Center Groningen.

a Post hoc analysis showed that the MDD-chronic group was significantly older than the MDD-remitted group (*p* < .005) and the MDD-improvement group (*p* = .01).

b Post hoc analysis showed that IDS scores at 2-year follow-up were significantly higher in the MDD-chronic group compared with the MDD-remitted (*p* < .001) and the MDD-improvement (*p* < .005) groups. IDS scores were also higher in the MDD-improvement group compared with the MDD-remitted group (*p =* .03).

c Post hoc analysis showed that the change in IDS scores from baseline to follow-up was significantly lower in the MDD-chronic group compared with the MDD-remitted group (*p* = .01).

**Table 2:** Balanced Prediction Accuracy (Sensitivity/Specificity) for All Classifiers Trained Separately for Whole-Brain Activation Patterns During the Faces Task, the Tower of London Task, Gray Matter Images, and Clinical Characteristics and Modalities Combined to Discriminate between MDD Subjects with Different Course Trajectories

Modality	MDD-CHR (*n* = 23) Versus	MDD-CHR (*n* = 23) Versus	MDD-IMP (*n* = 36) Versus
	MDD-REM (*n* = 59)	MDD-IMP (*n* = 36)	MDD-REM (*n* = 59)
Faces Task			
Angry > Baseline	64% (67/62)^a^	54% (53/55)	48% (42/54)
Fear > Baseline	62% (67/56)	59% (60/58)	40% (35/45)
Happy > Baseline	64% (73/54)^a^	69% (67/71)^a^	53% (55/51)
Sad > Baseline	58% (60/56)	49% (47/52)	45% (39/51)
Neutral > Baseline	53% (47/59)	67% (67/68)^a^	37% (32/41)
Overall Emotion > Baseline^b^	73% (80/67)^c^	59% (53/65)	50% (48/51)
Tower of London^d^	51% (53/50)	38% (37/46)	48% (46/50)
Gray Matter Images	43% (35/52)	53% (48/58)	43% (33/53)
Clinical Characteristics	69% (70/68)^a^	61% (61/61)	61% (69/53)
Faces Contrast Images and Clinical Characteristics Combined^e^	65% (52/78)^a^	52% (35/69)	54% (14/93)
All Modalities Combined f	62% (74/49)^a^	61% (65/57)	44% (43/44)

MDD-CHR, major depressive disorder chronic group; MDD-IMP, major depressive disorder gradual improvement in symptoms group; MDD-REM, major depressive disorder remitted group.

a *p* < .05 (corrected).

b Fusion of separate conditions based on the majority vote rule by counting the votes from the individual classifiers for the different emotional conditions. The class that receives the largest number of votes across emotional conditions is then selected as the class to which an individual belongs for the overall emotion condition and tested against the real class label.

c *p* < .01 (corrected).

d Based on brain activation patterns reflecting increasing task load (step 1 to step 5).

e Fusion of separate conditions based on the majority vote rule by counting the votes from the individual classifiers for the different emotional conditions and clinical characteristics. The class that receives the largest number of votes across emotional conditions and clinical characteristics is then selected as the class to which an individual belongs and tested against the real class label.

f Fusion of all modalities based on the majority vote rule by counting the votes from the individual classifiers for all different modalities. The class that receives the largest number of votes across modalities is then selected as the class to which an individual belongs based on all available data and tested against the real class label.
