# Supplementary material for: Predicting the Naturalistic Course of Major Depressive Disorder Using Clinical and Multimodal Neuroimaging Information: A Multivariate Pattern Recognition Study
Source: Biol Psychiatry. 2015 Aug 15;78(4):278–86. doi: 10.1016/j.biopsych.2014.11.018 (PMC4449319; doi:10.1016/j.biopsych.2014.11.018)
Supplement: Supplementary file 1 — Supplementary Material [file mmc1.pdf]

Predicting the Naturalistic Course of Major Depressive Disorder Using Clinical and Multimodal Neuroimaging Information: A Multivariate Pattern Recognition Study

Supplemental Information

Supplemental Methods

Exclusion Criteria NESDA-fMRI and Procedure

Exclusion criteria for the NESDA-MRI study were the presence of Axis I disorders other than MDD or anxiety disorder (i.e., panic disorder, social anxiety disorder and/or generalized anxiety disorder), use of psychotropic medication other than a stable use of selective serotonin reuptake inhibitors or infrequent benzodiazepine use, the presence or history of major internal or neurological disorder, dependency or recent abuse (past year) of alcohol or drugs, hypertension and the presence of MRI-contraindications.

Because the subgroup of participants from the total NESDA study that was included for MRI were scanned within 8 weeks after baseline assessment, the severity of symptoms (IDS scores) could have changed differently within the three groups from baseline to MRI assessment. We checked whether IDS scores at time of scanning were significantly different between the three course trajectory groups. The groups did not significantly differ from each other at time of scanning ($F_{(2,66)} = 1.69$, $p = 0.20$). There was a trend towards a main effect of time (slight decrease in symptoms between baseline and time of scanning in all groups; $F_{(1,66)} = 3.82$, $p = 0.06$), but no group by time interaction ($F_{(2,66)} = 0.77$, $p = 0.47$), indicating that the prognostic value of the neuroimaging methods for discriminating different course trajectories cannot be attributed to differences in depression state between the groups at time of scanning.

Task Paradigms

All task paradigms were programmed in E-prime software (Psychological Software Tools, Pittsburgh, PA).

Faces task

An emotional faces paradigm was used to assess brain activation during emotion processing. Color pictures of angry, fearful, sad, happy and neutral facial expressions, in addition to a control condition consisting of scrambled faces, selected from the Karolinska Directed Emotional Faces System (1) were presented. In an event-related design, 24 pictures were presented for each of five facial expressions (12 female and 12 male faces) in a pseudo-random presentation of a total of 200 pictures. Each face was not presented more than four times. The control condition (scrambled faces) was presented 80 times. Each picture was presented for 2.5 s, with an interstimulus interval (black screen) varying between 0.5 and 1.5 s. Subjects were asked to indicate each face’s gender with the index finger of the left or right hand. During the presentation of scrambled faces, participants had to press left or right buttons in conformity with the instruction presented on the screen (i.e., an arrow pointing to the left or to the right).

The mean reaction time to each of the five facial expressions was computed relative to the baseline (scrambled faces) condition.

Tower of London task

An event-related parametric version of the Tower of London task was used which consisted of 6 conditions: a baseline condition and five planning conditions ranging from one to five moves. In the planning trials, a starting configuration and a target configuration are presented. Each configuration presents three colored beads arranged on three pegs. Subjects were asked to work out the minimum number of times (ranging from 1 to 5) the beads in the starting configuration would have to be moved in order to make the arrangement of beads identical to that of the target configuration. One bead can be moved at a time and only when there is no other bead on top. Subjects could choose between two possible answers presented at the bottom left and right of the screen. Subjects had to indicate their answer by pressing the button corresponding to the side of the screen where the correct answer was presented. In the baseline condition, subjects were instructed to count the number of blue and yellow beads, requiring no planning activity. Specifically, the numbers of beads of each color in the two configurations, used for the baseline condition, were unequal, with the aim of preventing planning activity. We used a pseudo-randomized, self-paced design with a maximum response duration of 60 s for each trial. Each trial of three or more moves was followed by a baseline trial in order to control for any overflow effects (i.e., persevering of task-related cognitive processes after a difficult trial). No feedback regarding the answers was provided during the task. Both responses and response times were recorded and the proportion of correct answers was computed overall and per task load condition (baseline, 1 move, 2 moves, 3 moves, 4 moves, 5 moves).

Neuroimaging Data Acquisition Parameters

A gradient echo-planar sequence sensitive to blood oxygenation level-dependent contrast (TR = 2300 ms, TE = 30 ms [UMCG: TE = 28 ms], matrix size: 96 x 96 [UMCG: 64 x 64], voxel size: 2.29 x 2.29 mm in-plane resolution [UMCG: 3 x 3 mm], 35 slices [UMCG: 39], interleaved acquisition, 3 mm slice thickness) was used to acquire echo-planar images for each fMRI task. Three-dimensional T1-weighted images were collected using a gradient echo sequence (TR = 9 ms, TE = 3.5 ms, matrix size: 256 x 256, voxel size: 1 x 1 x 1 mm, 170 slices).

Structural Neuroimaging Data Preprocessing

The structural T1–images were normalized and segmented into gray matter (GM), white matter and cerebrospinal fluid using the voxel–based morphometry toolbox (VBM8; http://dbm.neuro.uni-jena.de/vbm.html) with default parameters. Preprocessing included bias-correction, tissue-classification using partial volume estimation and registration using an affine transformation and a nonlinear deformation using DARTEL. After pre-processing, the segmented images were spatially smoothed with an 8 mm Gaussian kernel. The ‘non-linear modulation only’ option was used to create volumetric GM partitions.

Functional Neuroimaging Data Preprocessing and Modelling

For the fMRI data, preprocessing included slice time correction, image realignment, co-registration of the functional images to the T1 scan, spatial normalization to Montreal Neurological Institute space as defined by the SPM8 T1-template, reslicing to 3 x 3 x 3 mm voxels, and spatial smoothing using an 8 mm Gaussian kernel. In a first level, single-subject fixed effects analysis, regressors were constructed by convolving trial onsets with a canonical hemodynamic response function and modulated in an event-related fashion. To account for low-frequency signal drift, a high-pass filter (1/128 Hz) was applied. Next, parameter estimates were generated for each condition. For the Faces task, contrast images were created for: angry > scrambled, fearful > scrambled, happy > scrambled, sad > scrambled and neutral > scrambled. The main effects of tasks across groups for each of the five contrasts based on a one sample *t*-test reported at a threshold of $p < 0.05$ whole brain corrected for family-wise error (PFWE_wholebrain) are presented in Figure S4. Replicating previous studies, viewing facial expressions (>scrambled faces) elicited fusiform gyrus and amygdala activation.

Contrast images for task load (with 1-5 move trials having weights [-1.5 -1 -0.5 1 2]) were calculated for the ToL task. The main effect of task load based on a one sample *t*-test reported at a threshold of $p < 0.05$ whole brain corrected for family-wise error (PFWE_wholebrain) is presented in Figure S5. Consistent with previous studies, brain activation in bilateral DLPFC, frontopolar regions, cingulate regions superior frontal regions, lateral parietal cortices, and precuneus increased with increasing task load.

Combining GP Classifiers

In addition to the classifiers described in the main text, a label fusion technique was applied to combine different data modalities into a single “consensus” classifier. The rationale behind this was that the different data modalities that are independently able to predict group depression course may classify subjects even more accurately when combined. Label fusion is a well-validated approach to combining distinct data sources (2) and is appealing for the present application because: (i) for neuroimaging data it is only slightly less accurate than state-of the art approaches based on learning a weighted combination of modalities, for example ‘multi-kernel learning’ approaches (3), whilst, (ii) not requiring that all subjects have data in all modalities (which would entail substantial reduction in sample size). A simple label fusion scheme was employed where each base classifier was assigned a ‘vote’ and the final class labels were assigned by taking the class with most votes with ties broken by a fixed rule (here, assigning ties to class one). We applied this voting procedure to combine all data modalities. For the analysis combining classifiers from different modalities, we allowed there to be a different number of modalities per subject (i.e., missing data was allowed). This means that as long as one modality was available, the subject was included in the analysis combining classifiers.

Permutation Testing for Statistical Inference

To assess the statistical significance of the balanced accuracy measures obtained from each classifier, a permutation testing procedure was performed. To achieve this, the labels were randomly permuted across subjects (1000 times) and the whole cross-validation procedure was repeated, storing the

balanced accuracy obtained from each permutation. A *p*-value for each classifier was obtained by counting the number of permutations for which the balanced accuracy from the permuted labels was greater than or equal to that obtained with the true (i.e., non-permuted) labels then dividing by 1000.

Predictive Maps and Statistical Parametric Maps

To characterize the discriminative pattern across brain regions, we first computed discriminative weights for each classifier, which describe the contribution of each voxel to the predictions. However, here we are more interested in the differential activity pattern across classes. Therefore, we converted these decoding weights to corresponding encoding models using the method presented in Haufe *et al.* (4). This yields a map with non-zero coefficients in every voxel that can be interpreted as quantifying differential regional effects. Since we are primarily interested in the pattern across all voxels, and not only voxels surviving an arbitrary threshold, we do not threshold these images.

To assist interpretation of the discriminative patterns, we also present the results from a standard mass-univariate analysis (i.e., a statistical parametric map). This was achieved by entering the contrast images from the first-level fixed effects models into a random effect model in SPM, where a *t*-contrast was then used to define the overall group difference. In the present work, the purpose of the SPMs are to assist interpretation of the multivariate maps, therefore we employed an exploratory threshold of $p < 0.001$ for visualization.

Supplemental Results

Task Performance

Task performance data were analyzed using the Statistical Package for the Social Sciences version 20 (SPSS Inc., Chicago, Illinois, USA). Data that were not normally distributed were first log transformed. Repeated measures ANCOVA were performed to examine group differences in task performance with condition (valence or task load) as a within-subject factor and group (MDD-REM, MDD-IMP, MDD-CHR) as a between-subjects factor. Performance scores on the ToL task and response times on the Faces task are depicted in Table S1. No effects of MDD course trajectory group or group by task load (ToL) or group by valence (Faces task) interaction effects on performance accuracy and response times were found. For the ToL task, we found a main effect of task load on response time ($F_{(4,103)} = 33.36$, $p < 0.001$), i.e., increasing response times with increasing task load. A trend towards a main effect of valence ($F_{(4,78)} = 2.34$, $p = 0.06$) was observed in the Faces task. Across groups, the reaction time was significantly slower to angry faces (relative to reaction time to scrambled faces) compared to all other conditions (all $p < 0.001$). In addition, the reaction time to sad faces was significantly slower than for neutral faces ($p = 0.02$).

GP Classification Results with Groups Matched on Age

Demographic and clinical characteristics are presented in Table S2. The different MDD course trajectory groups did not differ with regard to age, gender, years of education, scan location, antidepressant use at

baseline and follow-up, and IDS scores at baseline. With regard to IDS scores assessed at 2-year follow-up, the groups differed as expected ($F_{(2,66)}$ = 4.82, p = 0.01). IDS scores at follow-up were higher in the MDD-CHR group than MDD-REM ($t_{(44)}$ = 3.11, p = 0.003) and MDD-IMP ($t_{(44)}$ = 1.91, p = 0.05).

For the Faces task, fMRI data from eight MDD-CHR patients were discarded because of technical problems during scanning and for the ToL task, fMRI data from 4 MDD-CHR patients were discarded because of technical problems during scanning or poor performance (overall proportion correct responses <75%). This resulted in a sample size for each MDD group of n = 23 for gray matter, n = 15 for Faces task contrast images, n = 19 for ToL task contrast images and n = 23 for clinical characteristics.

GP Classification using Clinical Characteristics

Using baseline clinical information alone, the GP classifier did not discriminate between any of the course trajectories above chance level (Table S3).

GP Classification using Faces Task Contrast Images

The accuracies for discriminating between trajectories using neural activity patterns elicited by each type of facial expression are presented in Table S3.

Chronic (CHR) versus remitted (REM) patients

The GPCs for angry > scrambled faces, fearful > scrambled faces, happy > scrambled faces and sad > scrambled faces (but not neutral > scrambled faces) accurately discriminated between MDD-CHR and MDD-REM subjects.

Chronic (CHR) versus gradual improvement in symptoms (IMP) patients

Chronic subjects could be distinguished from the MDD-IMP subjects on basis of patterns of neural activity for happy > scrambled faces and neutral > scrambled faces (Table S3).

Gradual improvement in symptoms (IMP) versus remitted (REM) patients

Finally, the GPC discriminated between MDD-IMP and MDD-REM on basis of patterns of neural activity for sad > scrambled faces (Table S3).

GP Classification using Other Neuroimaging Modalities

The GPC did not discriminate between the MDD course trajectories above chance level using either patterns of neural activity in response to increasing task load of the ToL or gray matter images (Table S3).

Combining Classifiers from Different Modalities

The classifier combining all data modalities (n = 23 per group) discriminated between MDD-CHR and MDD-REM subjects and between MDD-CHR and MDD-IMP subjects (Table S3), with overall the highest

prediction accuracy (74% and 69%, respectively). The combined classifier did not discriminate above chance between MDD-REM and MDD-IMP subjects.

Whole Brain Predictive Maps and SPMs

Whole brain predictive maps and SPMs from the classifiers individually exceeding chance and discriminating between MDD-CHR and MDD-REM subjects are shown in Figure S2 and MDD-CHR and MDD-IMP subjects are shown in Figure S3. These show the same data as in Figures 2 and 3 in the main text, but show slices covering the whole brain.

Table S1. Performance of the different MDD course trajectory groups on the Faces task and the Tower of London task

**Paradigm**	**MDD-REM (*n* = 59)**	**MDD-IMP (*n* = 36)**	**MDD-CHR (*n* = 23)**
Faces Task			
Reaction time (ms)[a]			
angry	722.15 (132,94)	786.76 (169.47)	786.06 (193.74)
fearful	753.77 (138.60)	822.80 (199.14)	834.96 (211.55)
happy	769.32 (158.66)	836.13 (190.29)	848.28 (219.19)
sad	751.27 (130.48)	817.98 (156.09)	824.52 (210.95)
neutral	745.90 (146.74)	837.78 (190.43)	884.17 (259.28)
scrambled	683.02 (130.87)	725.35 (144.21)	818.77 (279.21)
Tower of London[b]			
Proportion correct:			
baseline	0.98 (0.04)	0.98 (0.03)	0.98 (0.02)
1 step	0.96 (0.06)	0.96 (0.06)	0.95 (0.05)
2 step	0.93 (0.11)	0.91 (0.13)	0.96 (0.05)
3 step	0.94 (0.09)	0.93 (0.12)	0.93 (0.09)
4 step	0.83 (0.16)	0.83 (0.20)	0.78 (0.18)
5 step	0.81 (0.16)	0.83 (0.18)	0.71 (0.26)
total	0.91 (0.07)	0.91 (0.09)	0.89 (0.09)

Data are given as mean (SD).

MDD-REM, major depressive disorder remitted group; MDD-IMP, major depressive disorder gradual improvement in symptoms group; MDD-CHR, major depressive disorder chronic group.

[a] A trend towards a main effect of valence ($F_{(4,78)} = 2.34$, $p = 0.06$), but no main effect of group ($F_{(2,81)} = 1.95$, $p = 0.16$) or group by valence interaction effect ($F_{(8,158)} = 0.89$, $p = 0.52$) was observed. Across groups, the reaction time was significantly slower to angry faces (relative to reaction time to scrambled faces) compared to all other conditions (all $p < 0.001$). In addition, the reaction time to sad faces was significantly slower than for neutral faces ($p = 0.02$).

[b] No main effect of task load ($F_{(4,103)} = 1.60$, $p = 0.18$), no main effect of group ($F_{(2,106)} = 0.01$, $p = 0.99$) and no group by task load interaction effect ($F_{(8,208)} = 0.94$, $p = 0.49$) was observed.

Table S2. Demographic and clinical characteristics of subjects included in the MVPA analyses with groups matched on age

**Characteristic**	**MDD-REM (*n* = 23)**	**MDD-IMP (*n* = 23)**	**MDD-CHR (*n* = 23)**	**Statistic**	***p* value**
Age, years	42.52 (8.86)	39.74 (7.55)	43.00 (10.24)	$F = 0.82$	0.42
Gender, *n* (%)					
Female	17 (74)	13 (57)	13 (57)	$X^2 = 1.98$	0.37
Male	6 (26)	10 (43)	10 (43)		
Education, years	11.96 (3.54)	12.17 (2.86)	12.48 (2.54)	$F = 0.18$	0.84
Scan location, *n* (%)					
AMC Amsterdam	9 (39)	8 (35)	9 (39)	$X^2 = 0.18$	0.99
LUMC Leiden	8 (35)	8 (35)	8 (35)		
UMCG Groningen	6 (26)	7 (30)	6 (26)		
IDS total T1	32.04 (9.99)	33.61 (9.39)	35.78 (8.28)	$F = 0.95$	0.39
IDS total T2	21.00 (8.80)	24.09 (9.80)	29.70 (10.13)	$F = 4.86$	0.01[a]
IDS change (T2-T1)	-11.04 (10.91)	-9.52 (10.76)	-6.08 (9.82)	$F = 1.34$	0.27
Antidepressant use T1, *n* (%)					
No	13 (57)	14 (61)	14 (61)	$X^2 = 0.12$	0.94
Yes	10 (43)	9 (39)	9 (39)		
Antidepressant use T2, *n* (%)					
No	13 (57)	16 (70)	15 (65)	$X^2 = 0.88$	0.65
Yes	10 (43)	7 (30)	8 (35)		

Data are given as mean (SD).
MDD-REM, major depressive disorder remitted group; MDD-IMP, major depressive disorder gradual improvement in symptoms group; MDD-CHR, major depressive disorder chronic group; AMC, Academic Medical Center; LUMC, Leiden University Medical Center; UMCG, University Medical Center Groningen; IDS, Inventory of Depressive Symptoms; T1, baseline; T2, 2-year follow-up.
[a] Post-hoc analysis showed that IDS scores at 2-year follow-up was significantly higher in the MDD-chronic group compared to the MDD-remitted ($p = 0.003$) and the MDD-improvement ($p = 0.05$) groups.

Table S3. Balanced accuracy (sensitivity/specificity) for all classifiers trained separately for whole brain activation patterns during the Faces task, the Tower of London task, grey matter images and clinical characteristics and all modalities combined to discriminate between MDD subjects with different course trajectories, with groups matched on age.

**Modality**	**MDD-CHR versus MDD-REM**	**MDD-CHR versus MDD-IMP**	**MDD-DEC versus MDD-IMP**
Faces Task			
Angry > baseline	67%* (73/60)	37% (36/38)	47% (53/40)
Fear > baseline	67%* (67/67)	57% (57/56)	40% (40/40)
Happy > baseline	67%* (67/67)	67%* (67/67)	43% (40/47)
Sad > baseline	67%* (67/67)	40% (40/40)	73%** (73/73)
Neutral > baseline	50% (47/53)	67%* (65/69)	53% (47/60)
Overall emotion > baseline[a]	70%* (80/60)	60% (60/60)	50% (53/47)
Tower of London[b]	40% (37/42)	47% (32/63)	50% (42/58)
Gray matter images	41% (43/39)	43% (39/48)	46% (52/39)
Clinical characteristics	59% (61/57)	59% (57/61)	41% (57/26)
All modalities combined[c]	74%** (74/75)	69%* (61/78)	41% (19/65)

* $p < 0.05$.
** $p < 0.01$.
MDD-REM, major depressive disorder remitted group; MDD-IMP, major depressive disorder gradual improvement in symptoms group; MDD-CHR, major depressive disorder chronic group.
[a] Fusion of separate conditions based on the majority vote rule by counting the votes from the individual classifiers for the different emotional conditions. The class which receives the largest number of votes across emotional conditions is then selected as the class to which an individual belongs for the overall emotion condition and tested against the real class label.
[b] Based on brain activation patterns reflecting increasing task load (step 1 to step 5).
[c] Fusion of all modalities based on the majority vote rule by counting the votes from the individual classifiers for all different modalities. The class which receives the largest number of votes across modalities is then selected as the class to which an individual belongs based on all available data and tested against the real class label.

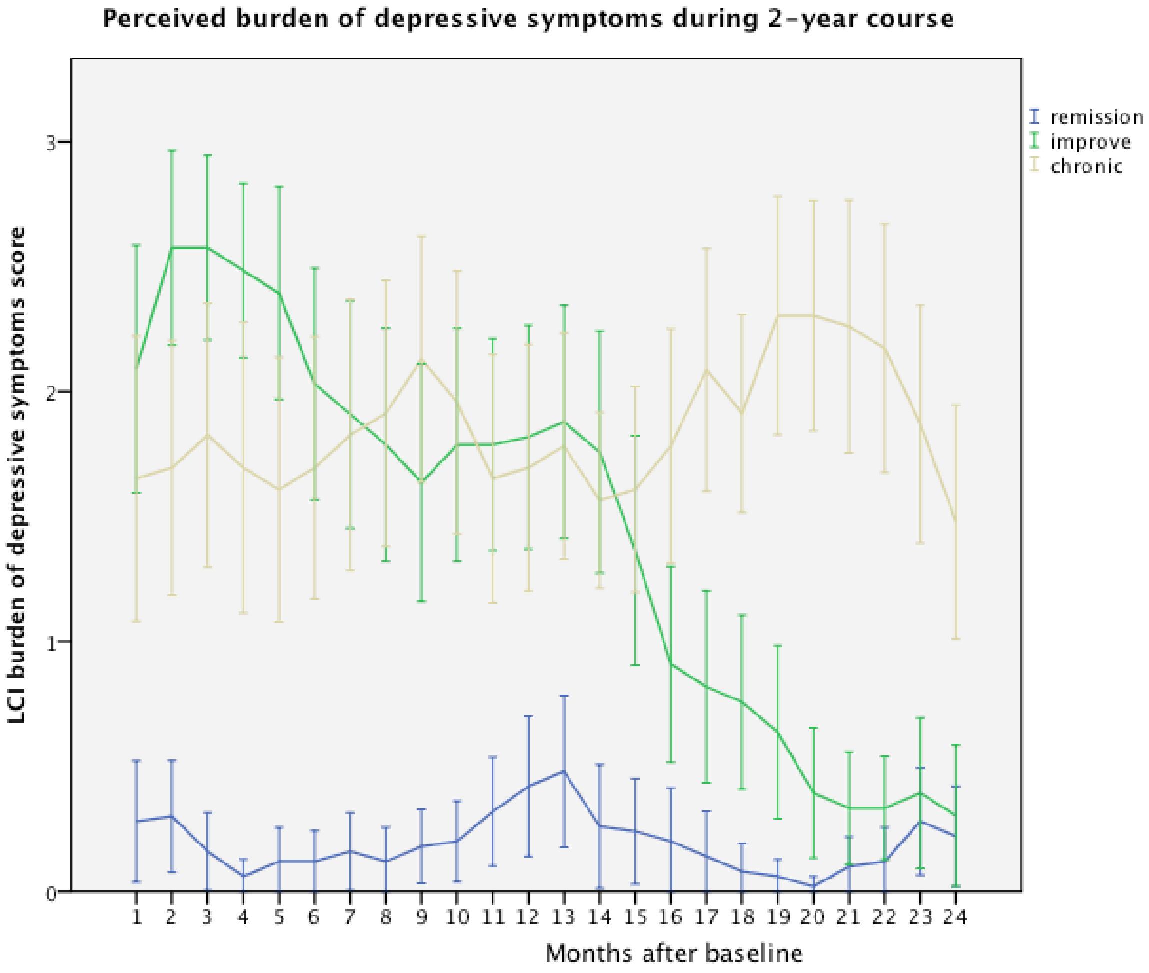

Figure S1. Perceived burden of depressive symptoms score (mean ± SE; ranging 0-5) during the 24-month follow-up period for each of the three course trajectories (green: MDD-remitted, showing a rapid remission of symptoms after baseline assessment (*n* = 59), blue: MDD-improve, showing a gradual decline of symptoms from baseline to follow-up (*n* = 36), brown: MDD-chronic, showing no relief from symptoms from baseline to follow-up.

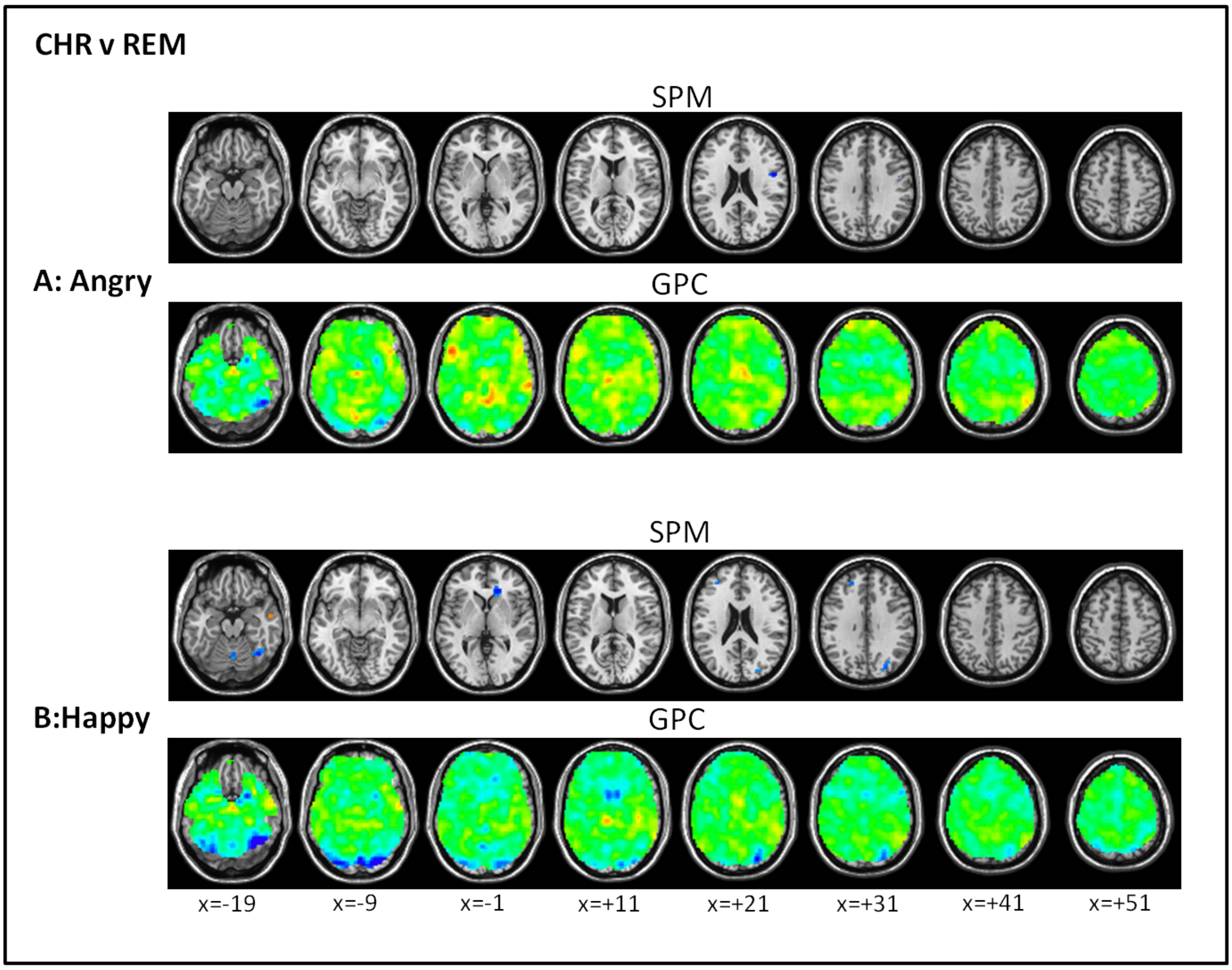

Figure S2. Whole brain GPC predictive maps discriminating MDD-CHR from MDD-REM subjects plus statistical parametric maps (SPMs; thresholded at $p < 0.001$), presented separately for the contrasts (**A**) angry versus scrambled faces and (**B**) happy versus scrambled faces. The red colors indicate higher prognostic value for the first class (i.e., MDD-CHR) and blue colors indicate voxels with a higher prognostic value for the second class (MDD-REM). MDD; major depressive disorder, CHR; chronic, REM; remitted.

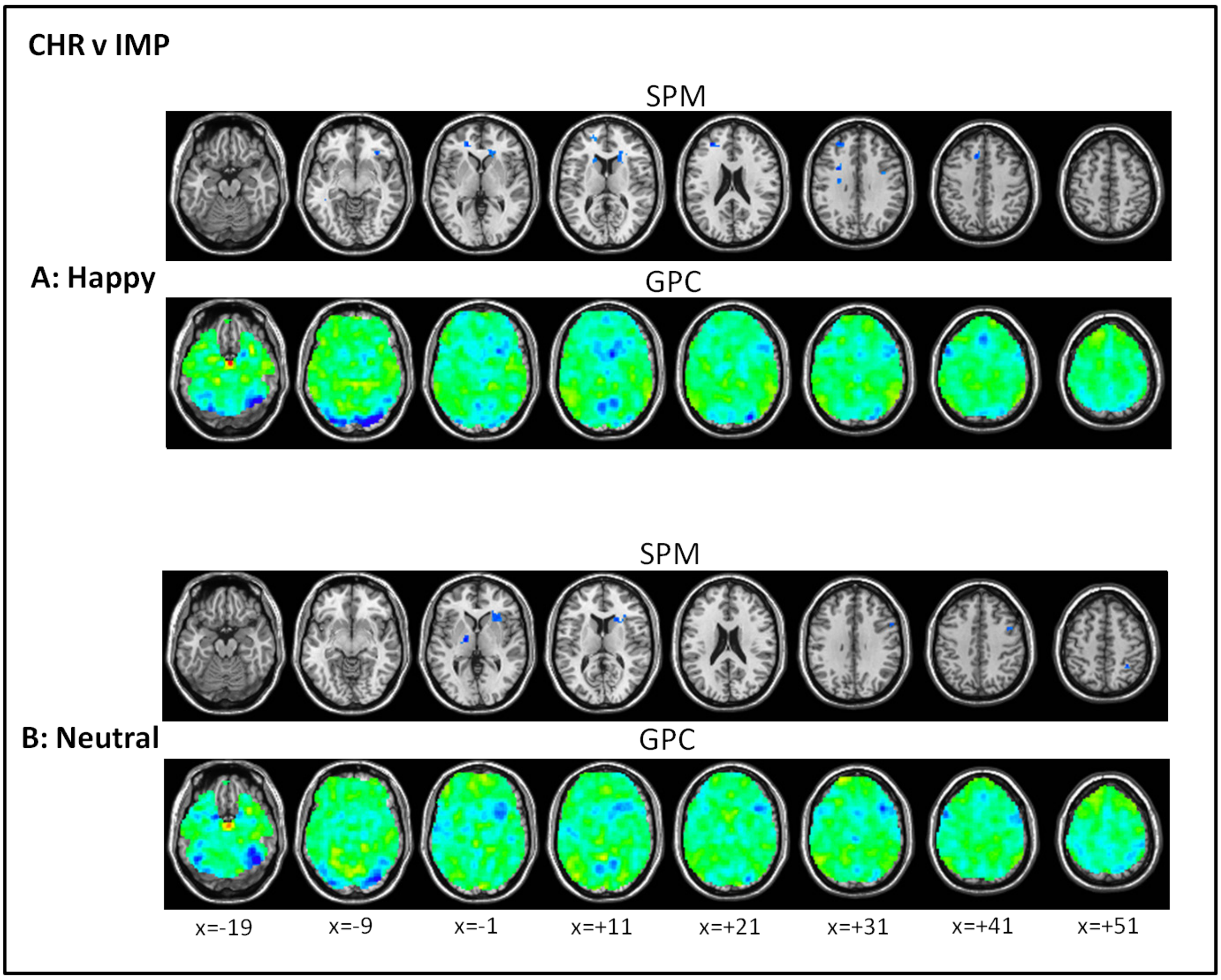

Figure S3. Whole brain GPC predictive maps discriminating MDD-CHR from MDD-IMP subjects, and statistical parametric maps (SPMs; thresholded at $p < 0.001$) presented separately for the contrasts (**A**) happy versus scrambled faces and (**B**) neutral versus scrambled faces. The red colors indicate higher prognostic value for the first class (i.e., MDD-CHR) and blue colors indicate voxels with a higher prognostic value for the second class (MDD-IMP). MDD; major depressive disorder, CHR; chronic, IMP; improved.

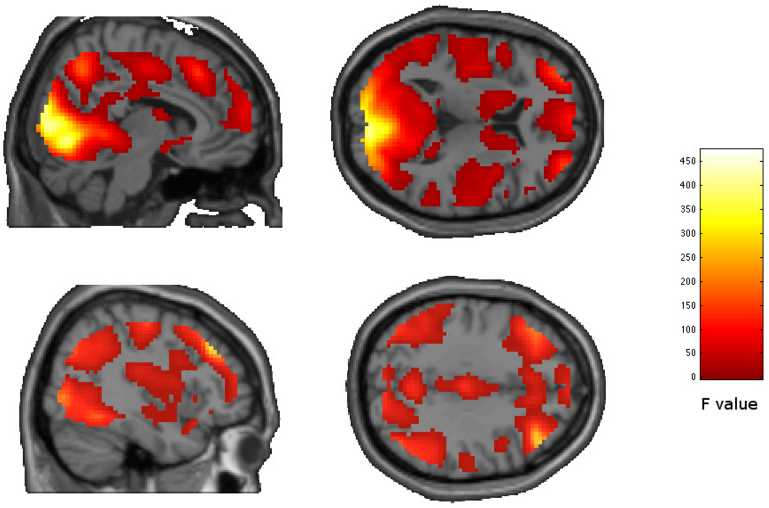

Figure S4. Main effect of task load in the Tower of London task across groups ($p < 0.05$ whole brain FWE corrected).

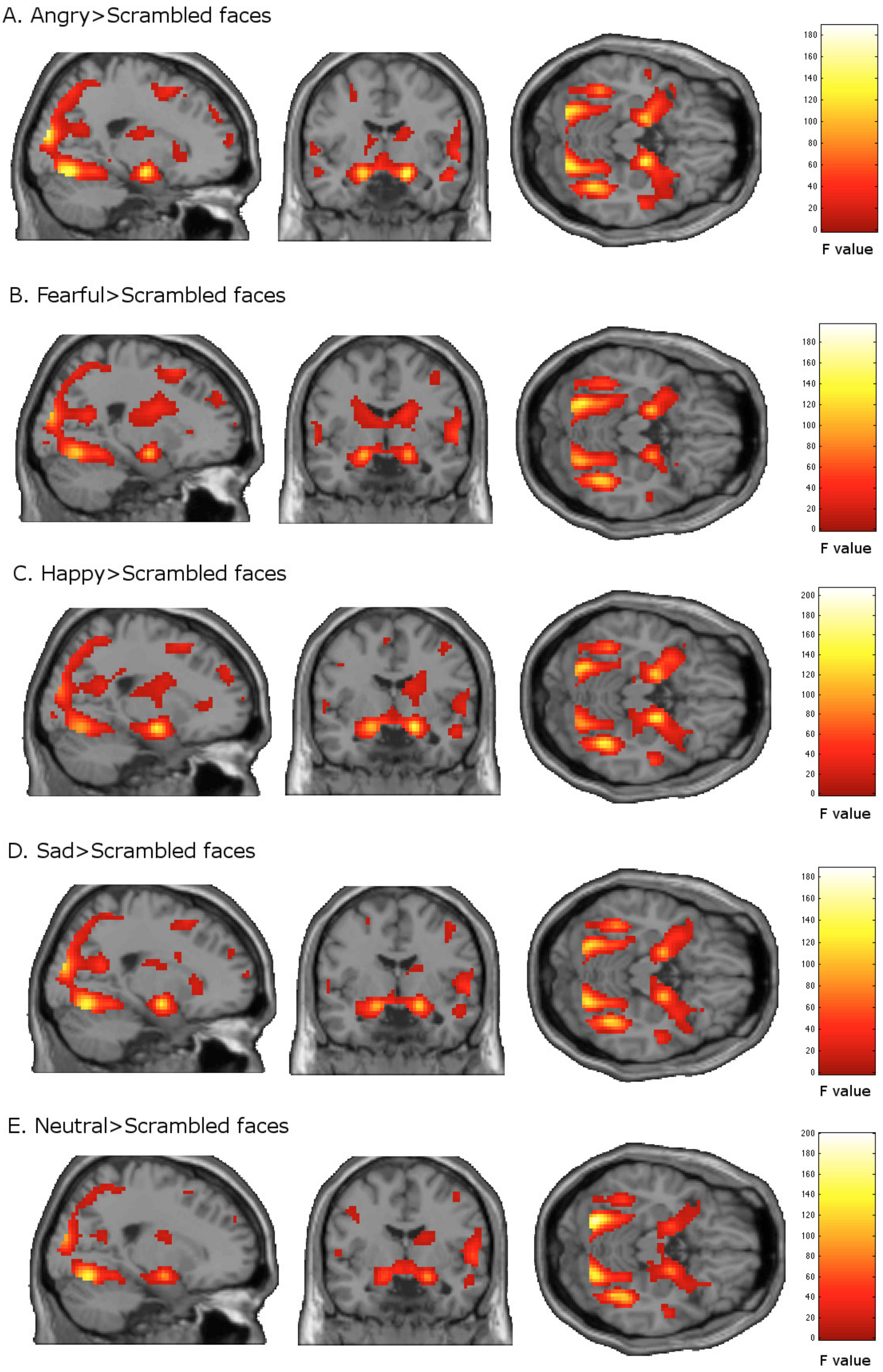

Figure S5. Main effect of emotional facial expressions across groups ($p < 0.05$ whole brain FWE corrected), separate for the (**A**) angry > scrambled, (**B**) fearful > scrambled, (**C**) happy > scrambled, (**D**) sad > scrambled and (**E**) neutral > scrambled conditions.

Supplemental References

1. Lundqvist D, Flykt A, Ohman A (1998): *The Karolinska Directed Emotional Faces.* CD ROM from Department of Clinical Neuroscience,Psychology Section,Karolinska Institute.
2. Kittler J, Hatef M, Duin RPW, Matas J (1998): On Combining Classifiers. *IEEE Trans Pattern Anal Mach Intell* 20:3.
3. Zhang D, Shen D; Alzheimer's Disease Neuroimaging Initiative (2012): Multi-modal multi-task learning for joint prediction of multiple regression and classification variables in Alzheimer's disease. *Neuroimage* 59:895-907.
4. Haufe S, Meinecke F, Görgen K, Dähne S, Haynes JD, Blankertz B, Bießmann F (2014): On the interpretation of weight vectors of linear models in multivariate neuroimaging. *Neuroimage* 87:96-110.
